# Numerical analysis of pore-scale CO_2_-EOR at near-miscible flow condition to perceive the displacement mechanism

**DOI:** 10.1038/s41598-023-39706-1

**Published:** 2023-08-03

**Authors:** Parisa Behnoud, Mohammad Reza Khorsand Movaghar, Ehsan Sabooniha

**Affiliations:** 1 Department of Petroleum Engineering, Amirkabir University of Technology (Tehran Polytechnic), P.O. Box 15875-4413, 424 Hafez Avenue, Tehran, Iran 1591634311; 2 DTU offshore, Technical University of Denmark, Copenhagen, Denmark

**Keywords:** Carbon capture and storage, Fluid dynamics, Chemical engineering

## Abstract

Gas flooding through the injection of $\text{CO}_{2}$ is generally performed to achieve optimum oil recovery from underground hydrocarbon reservoirs. However, miscible flooding, which is the most efficient way to achieve maximum oil recovery, is not suitable for all reservoirs due to challenge in maintaining pressure conditions. In this circumstances, a near-miscible process may be more practical. This study focuses on pore-scale near-miscible $\text{CO}_{2}$–Oil displacement, using available literature criteria to determine the effective near-miscible region. For the first time, two separate numerical approaches are coupled to examine the behavior of $\text{CO}_{2}$–oil at the lower-pressure boundary of the specified region. The first one, the Phase-field module, was implemented to trace the movement of fluids in the displacement $\text{CO}_{2}$–Oil process by applying the Navier–Stokes equation. Next is the TDS module which incorporates the effect of $\text{CO}_{2}$ mass transfer into the oil phase by coupling classical Fick’s law to the fluids interface to track the variation of $\text{CO}_{2}$ diffusion coefficient. To better recognize the oil recovery mechanism in pore-scale, qualitative analysis indicates that interface is moved into the by-passed oil due to low interfacial tension in the near-miscible region. Moreover, behind the front ahead of the main flow stream, the $\text{CO}_{2}$ phase can significantly displace almost all the bypassed oil in normal pores and effectively decrease the large amounts in small pores. The results show that by incorporating mass transfer and capillary cross-flow mechanisms in the simulations, the displacement of by-passed oil in pores can be significantly improved, leading to an increase in oil recovery from 92 to over 98%, which is comparable to the result of miscible gas injection. The outcome of this research emphasizes the significance of applying the $\text{CO}_{2}$-EOR process under near-miscible operating conditions.

## Introduction

$\text{CO}_{2}$ Gas flooding has long been regarded as a popular method of improving oil recovery and many approaches have been proposed to optimize gas injection systems^1–5^. $\text{CO}_{2}$ injection has been extensively used in the oil industry for many years as an EOR method^6,7^. While $\text{CO}_{2}$-based EOR can improve oil recovery by reducing oil viscosity and decreasing mobility of $\text{CO}_{2}$, it is of paramount importance for reducing gas emissions and carbon storage and $\text{CO}_{2}$ sequestration applications as well^8–10^. Moreover, recently geological $\text{CO}_{2}$ capture and storage of flue gas in hydrate reservoirs have been investigated by putting a significant amount of $\text{CO}_{2}$ underground for tonnes of hydrocarbon (methane) produced which is in the same vein as the studies to achieve net zero^11,12^.

Moreover, sensitivity analysis was implemented to investigate the effect of seven reservoir parameters, namely reservoir porosity, horizontal permeability, temperature, formation stress, the ratio of vertical to horizontal permeability, capillary pressure, and residual gas saturation on geological CO_2_ storage capacity.

Note that the ratio of vertical to horizontal permeability or anisotropy ratio is paid attention to which the results are as follows.

The sensitivity of the factors affecting the gas capture capacity of CO_2_ decreases in the order of formation stress, temperature, residual gas saturation, horizontal permeability, and porosity^13^**.**

In this regard, another study was carried out by combining a comprehensive large-scale 3D reservoir simulation by running single-porosity, dual-permeability, dual-porosity models and a computation-efficient DACE technique ("Design and Analysis of Computer Experiments") to analyze the sensitivity of CO_2_ storage in fractured aquifers.

The dynamic model comprehensively simulated CO_2_ storage performance in aquifers by considering all trapping mechanisms except for mineralization, such as structural, dissolution, residual, and local capillary trapping.

The major outcome of this study demonstrate that fractures play a negative role on CO_2_ trapping. Thus, it is necessary to create dual permeability/dual-porosity models to generate realistic prediction^14^.

In contrast to Continuous Gas Injection (CGI) and Water-Alternating-Gas (WAG) injection methods, Gas-Assisted Gravity Drainage (GAGD) relies on the natural separation of reservoir fluids to achieve stable oil displacement through gravity. The process involves injecting gas through vertical wells to create a gas cap that allows oil and water to drain down to horizontal producers, resulting in improved oil recovery. The CO_2_-GAGD process have been payed more attention in recent years. Consequently, several studies have been performed as immiscible injection modes were used to implement the CO_2_-GAGD process and enhance oil recovery in a specific section of the main pay/upper sandstone member in the South Rumaila oil field in Iraq. To optimize future oil recovery via the CO_2_-GAGD process, Design of Experiments (DoE) and Proxy Modeling techniques were employed via Equation of state compositional field/Reservoir Simulation^15–19^.

To optimize and evaluate $\text{CO}_{2}$ injection process, it is crucial to understand $\text{CO}_{2}$–oil flow behavior in porous media.

There have been studies on the sensitivity analysis of effective parameters on CO_2_–Oil flow behavior and the corresponding rate of production.

Recent studies by Al-Mudhafar et al. have discussed this issue, investigating the weight of sensitivity analysis on three reservoir parameters, namely porosity, permeability (vertical), and anisotropy ratio of permeability. The results show that permeability is the most important parameter in all reservoir layers. Although anisotropy ratio moderately influences the production layers, especially the water zone, the effect has been absent in transition and injection layers of the reservoir. In the CO_2_-GAGD gas injection process, porosity has not influenced oil recovery in all reservoir layers^20–22^.

There has been a lot of arguments in the literature on the promising effect of miscibility and near-miscibility conditions during $\text{CO}_{2}$ flooding^23–25^. However, fully miscible flooding is considered a very expensive and difficult approach in terms of economic and operational standpoints due to high costs of providing rich gas injectant and reaching high-pressure injection condition. Additionally, it might not lead to proper additional oil recovery improvement in comparison with near-miscible condition.

Moreover, to improve the productivity of a (single) well, another study investigated the effect of miscible gas injection with geomechanical effects in tight reservoirs during CO_2_-prepad injection before hydraulic fracturing.

The results show that among these parameters, bottom-hole flow pressure, reservoir thickness, and fracture conductivity had the greatest effect on cumulative production. Although bottom-hole flow pressure and fracture conductivity are controllable factors, bottom-hole flow pressure (which can be adjusted and manipulated) is the most influential parameter.

Therefore, generally $\text{CO}_{2}$ near-miscible flooding is preferred as a more feasible alternative way^26–29^.

An injection of near-miscible gas consists of injecting gases that do not develop complete miscibility with the oil, but are rather close to it^30^. Bui et al. showed that at near miscible conditions, oil extraction in not the only mechanism of mass transfer between hydrocarbon components and $\text{CO}_{2}$. They illustrated that the viscosity reduction of oil phase due to dissolving $\text{CO}_{2}$ into oil phase also dramatically contributes to additional recovery factor as the extraction mechanism^31^. There have been several works in the literature to predict a reasonable pressure interval for $\text{CO}_{2}$ near-miscible displacement^32–34^. Very recently, Chen et.al introduced some empirical correlations to predict minimum miscibility pressure and the effective near miscible pressure region for both pure and impure $\text{CO}_{2}$ injection projects which can be applicable to every specific reservoir. **Hence, the region is defined from lower limit as 0.87 MMP to upper limit as 1.07 MMP.** This work can provide a practical tool for characterizing near-miscible region and designing future near-miscible $\text{CO}_{2}$ floods^35^. 34, most of the researches in the literature generally investigate $\text{CO}_{2}$–Oil displacement on core-scale and field-scale works and there are a few studies in the literature that focus on pore-scale investigation of $\text{CO}_{2}$–oil complex behavior at different conditions. Pore-scale studies are considered robust approaches for visualization of fluids displacement mechanisms, characterizing micro-scale fluid–fluid and fluid/rock interactions, and analyzing fluids distribution profiles with respect to effective forces at micro-scale^36–39^. In this regard, Huang et al. evaluated $\text{CO}_{2}$ exsolution in $\text{CO}_{2}$ huff-n-puff procedure for EOR and $\text{CO}_{2}$ storage applications. They showed that initial state of near-miscible $\text{CO}_{2}$–oil would lead to intense $\text{CO}_{2}$ nucleation. They also emphasized that presence of water can increase $\text{CO}_{2}$ saturation in the system to 95% regardless of the wettability^40^. Seyyedi et al. investigated multi-phase flow of $\text{CO}_{2}$-water–oil system in a high-pressure micro-model at near-miscible condition. They indicated that despite low sweep efficiency of $\text{CO}_{2}$–Oil displacement at initial stages of injection due to high $\text{CO}_{2}$ mobility, the diffusion of $\text{CO}_{2}$ into the oil phase can cause capillary crossflow across the trapped oil and improve the recovery factor after breakthrough time. Their obtained results illustrates the importance of $\text{CO}_{2}$ diffusion at near-miscible $\text{CO}_{2}$ floods^41^. Zhu et al. studied the drainage process of $\text{CO}_{2}$–oil system in an oil-wet porous media using phase-field interfacing capturing method. By performing wide range of sensitivity analysis over gravity number, capillary number and viscosity ratios, they depicted that viscous force is the dominant mechanism during $\text{CO}_{2}$-EOR procedure, and when viscous force is small, gravity fingers improve the sweep efficiency of $\text{CO}_{2}$–oil displacement. They also illustrated that after $\text{CO}_{2}$ breakthrough, the pressure in the main $\text{CO}_{2}$ flow path dramatically decreases, and the oil phase to re-flows into large pores previously occupied by $\text{CO}_{2}$^42^. Ma et al., recently performed a numerical study on immiscible, near-miscible and miscible flooding using different approaches. Their results indicated that while near-miscible flooding is more favorable in terms of sweep efficiency compared to immiscible flooding, it is still not able to displace oil in smaller pore throats. They expressed that $\text{CO}_{2}$ diffusivity effect is negligible during miscible flooding. It is worth mentioning that in their work, mas transfer mechanism is completely ignored for near-miscible flooding, and interfacial tension is assumed to be constant during the whole simulations^43^.

In the current study, we exclusively focus on pore-scale near-miscible $\text{CO}_{2}$ flooding and investigate the behavior of $\text{CO}_{2}$–oil flow at different pressures in near-miscible pressure interval since this interval is more economically and operationally demanding. At first, minimum miscibility pressure (MMP) and lower pressure boundary limit was calculated for the presented system to characterize the effective near-miscible flooding region where interfacial tension between oil and $\text{CO}_{2}$ has not fully disappeared and near miscible effects associated with $\text{CO}_{2}$ flooding is dominant^35^. Then a sensitivity analysis is done to investigate the oil recovery factor at two different pressures in effective near-miscible region. The novelty of the current work lies in incorporating $\text{CO}_{2}$–oil mas transfer at the interface to further characterize the important near miscible mechanism including oil condensation/vaporization. For the first time, to model the movement of fluids in the displacement CO_2_–Oil process by applying Navier–Stokes equation and incorporating the effect of mass transfer at the interface of two fluids and the diffusion of carbon dioxide into oil by implementing classical Fick's law, the phase field and TDS modules respectively and simultaneously have been coupled with each other in pore scale studies. Additionally, dynamic interfacial tension (IFT) and diffusion coefficient variation is studied to understand the effect of pressure gradient on diffusive interface parameters in a $\text{CO}_{2}$-flooding system. The obtained results demonstrate the significance of $\text{CO}_{2}$ mass transfer in near-miscible floods along which cannot be ignored. The current research also proposes an optimum criterion in designing $\text{CO}_{2}$ near miscible flooding which can be helpful in $\text{CO}_{2}$-EOR application.

The main parts of the introduction section are presented concisely and separately in Table 1 based on the topic they have addressed to easily follow the underlying logic in this section.

**Table 1:** Consice information about literature review.

Subject	References
First Paragraph	
Significance of the study	^1–12^
Gas Storage	
CO_2_ Storage	^8–12^
CO_2_ Storage Sensitivity Analysis	^13,14^
Gas Flooding	
Review on different gas Injection Processes	^15,18,19^
CO_2_- Gas Assisted Gravity Drainage (GAGD)	^15–17^
CO_2_-GAGD Sensitivity Analysis	^20–22^
CO_2_-Prepad injection	
Effect of geomechanical and miscibility in the gas injection process	^29^
Near-Miscible injection	
Near-miscible mechanism	^31^
Pressure interval for CO_2_ near-miscible displacement	^32–35^
Near-miscible pore scale/micro model studies	^36–43^
Last Paragraph	
Problem Statement	This study
Novelty	
Procedures and methods	

## Theory and numerical approach

The numerical method for this study is represented by an isothermal two-phase flow in the heterogenous porous media where the properties of the oil phase and diffusive interface dynamically changes due the alteration of $\text{CO}_{2}$ concentration and pressure in the system respectively. For this purpose, COMSOL Multiphysics of version 5.6 was chosen which is a finite element analysis, solver, and simulation software package for various physics and engineering applications, especially coupled phenomena and multiphysics^44^. This software facilitates conventional physics-based user interfaces and coupled systems of partial differential equations (PDEs). COMSOL provides the interdigitated electrodes (IDEs) and unified workflow for electrical, mechanical, fluid, acoustics, and chemical applications.

In this software, Navier–Stokes momentum equations are coupled with Phase Field method for immiscible $\text{CO}_{2}$ and oil phase, and The Transport of Diluted Species Interface (TDS) method to account for diffusive interface between miscible $\text{CO}_{2}$ mass transfer at the same time. TDS method is used to calculate the concentration field of a dilute solute in a solvent. Transport and reactions of the species dissolved in a gas, liquid, or solid can be handled with this interface. The driving forces for transport can be diffusion by Fick’s law, convection when coupled to a flow field, and migration, when coupled to an electric field^44^.

Governing equations, numerical scheme and computational geometry are described in the following section.

### Model geometry

**Figure 1:**
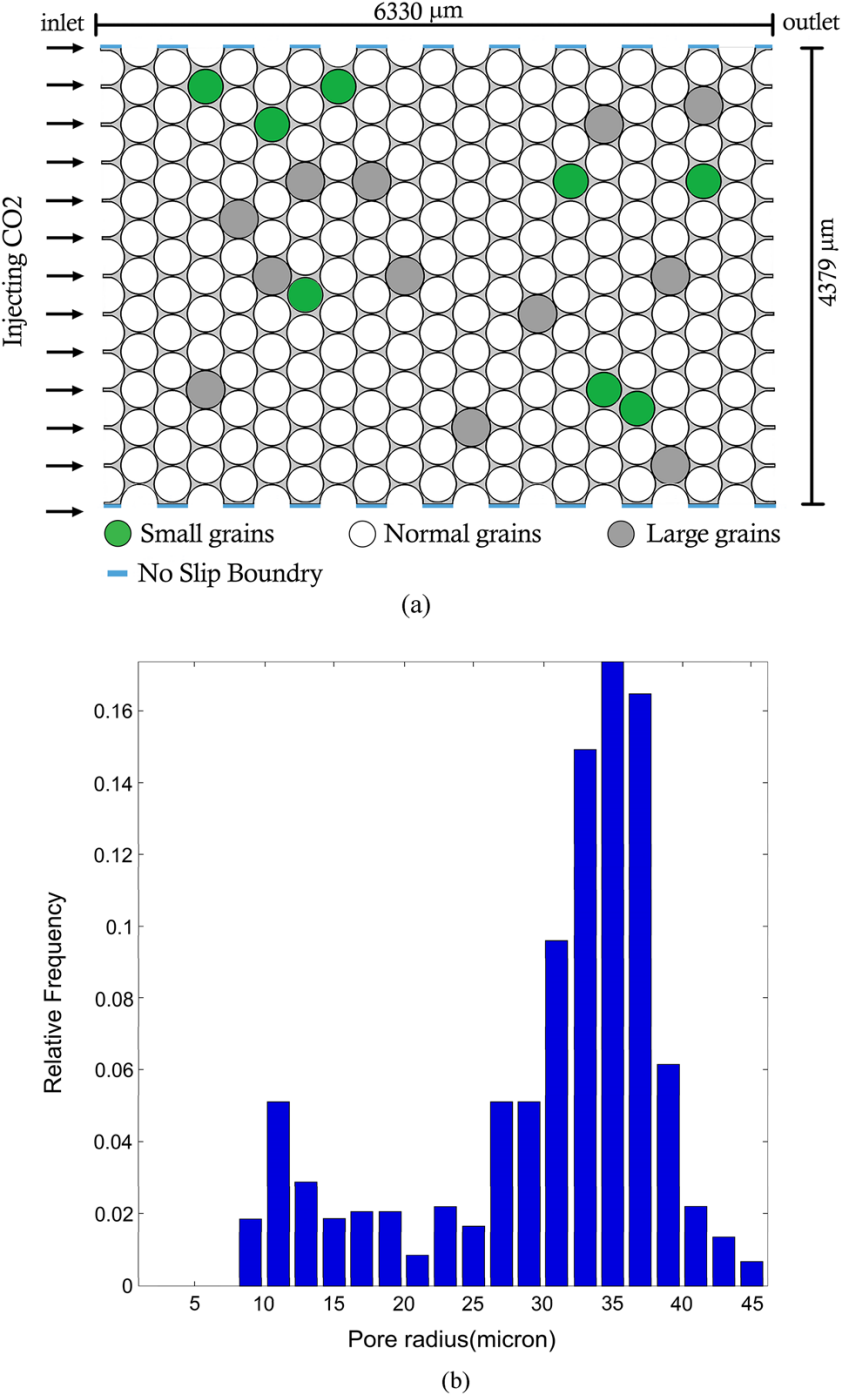
(**a**) Computational domain geometry. $\text{CO}_{2}$ Enters the medium from the left side and exits from the right side. The black area represents the porous media and the matrix grains are shown with gray color. (**b**) Distribution of pores in the model.

The computational domain in this study is a heterogenous porous media with dimension of $6330 \times 4379$ $\mathrm{\mu m}$ which consists of several circular-shape grains with a diameter of $350 \mathrm{\mu m}$^43^. In this model, the diameters of twenty random grains are either reduced or enlarged by 5% to include heterogeneity effect. The green color grains are the grains with reduced diameter and the grey grains represent the enlarged ones (Fig. 1a). Figure 1b also illustrates the distribution of pore sizes in the selected porous media. The detailed characteristics of the simulated domain are further elaborated in Table 2.

**Table 2:** Properties of the computational domain.

Avg. pore diameter (${\mu m}$)	Avg. Grain Size (${\mu m}$)	Porosity	Absolute Permeability (Darcy)
30.47	350	0.35	2

### Boundary conditions and initial values

In an attempt of modelling near-miscible flooding condition throughout the whole computational space, the displacing $\text{CO}_{2}$ phase will be injected into the medium, which had previously been saturated with oil, with constant pressure of ${P}_{inj}$, from the left-hand side. The pressure on the right-hand side of the porous medium will be set on ${P}_{out}$, as well. In this study, the minimum miscibility pressure (MMP) and the lower boundary of effective near-miscibility pressure zone are assessed from empirical equation to be equal to 12.7 MPa and 11.05 MPa, respectively^35^. Accordingly, the ${P}_{inj}$ and ${P}_{out}$ were set on the values of 11.05 + ε MPa and 11.05 MPa, respectively. It is noteworthy to mention, the initial pressure of the system ${P}_{init}$ was set to the value of 11.05 MPa (lower limit of effective near-miscible region). The pressure difference between the inlet and outlet should be small enough to provide a sensible two-phase flow/displacement in the pore scale. The parameter ɛ is set to 600 *Pa* $\left(\cong 0.1 Psi\right)$, accordingly. The opted value for ε is consistent with the dimensions of the system, with pressure drop of 1 Psi, as well as Danesh et al. study on near-miscible injection of methane gas in decane model oil in a lab micromodel^28^. As a result, one can compare the results of the aforementioned system with commercial EOR/IOR flooding program designs. The wetted wall boundary condition is selected on the particle grain surfaces with a constant contact angle (θ = $\frac{\pi }{6}$).

### Governing equations

The flow regime is assumed to be laminar while the fluids are supposed to be Newtonian and incompressible. Gravity is neglected and the fluids displacement will be investigated at 2D scale.

In order to separate two phases by a fluid–fluid diffusive interface, Cahn–Hilliard phase-field method^45^ coupled with Navier–Stokes and continuity equations were employed. In phase-field model, which is based on the minimum of free energy principle, Ginzburg–Landau equation is implemented to calculate the mixing energy^46,47^:

1 $${f}_{mix}\left(\varphi ,\nabla \varphi \right)= \frac{1}{2} \lambda {\left|\nabla \varphi \right|}^{2}+ \frac{\lambda }{4{\varepsilon }^{2}} {({\varphi }^{2}-1)}^{2}$$

The minimization of the gradient component (first term on the left-hand side) leads to the phases mixing, and the minimization of double well potential (the second term on the right-hand side) causes phase separation.

Unitless phase-field parameter $(\varphi )$ is used to determine the relative concentration of each phase. In this regard, $-1< \varphi <1$ depicts the interface area and $\varphi =\pm 1$ illustrates the pure phases. The volume fraction of phases is then described by $(1+\varphi )/2$ and $(1-\varphi )/2$ equations which define the fluid properties in the system^48,49^.

2 $$\vartheta \left(\varphi \right)= \frac{(1+\varphi )}{2}{\vartheta }_{1}+ \frac{(1-\varphi )}{2} {\vartheta }_{2}$$

where $\vartheta$ is a component property (e.g., viscosity). The Navier–Stokes equation is modified by including continuity equation and adding a phase-field dependent surface force to capture the moving interface^49,50^. In the current project, it is assumed that $\text{CO}_{2}$ and oil ideally mix with each other and during the injection and no chemical reaction takes place. As the result, to incorporate the $\text{CO}_{2}$–oil mass transfer and cross over flow at the interface, classical Fick’s law was implemented^51^. The main governing equations of Cahn–Hilliard phase-field coupled with Navier–Stokes and convective-diffusion mas transfer are presented here:

3 $$\rho \frac{\partial {\varvec{u}}}{\partial t}+ \rho \left({\varvec{u}}.\nabla \right){\varvec{u}}= -\nabla p+\nabla .\left[\mu \left(\nabla {\varvec{u}}+\nabla {{\varvec{u}}}^{T}\right)\right]+G\nabla \varphi$$

4 $$\nabla .\boldsymbol{ }{\varvec{u}}=0$$

5 $$\psi = -\nabla .{\varepsilon }^{2}\nabla \varphi +({\varphi }^{2}-1)\varphi$$

6 $$\frac{\partial c}{\partial t}+{\varvec{u}}.\nabla c= \nabla . D\nabla c$$

where* t* denotes the time, p is pressure, ***u*** is the fluid velocity field, c is the concentration of $\text{CO}_{2}$ phase, D depicts diffusion coefficient. The auxiliary parameter $\psi$ decomposes the Cahn–Hilliard equation into two separate equations. *γ* denotes the mobility parameter, *ε* defines the thickness of the interface, and $\lambda$ is the mixing energy density. The chemical potential $G$ is $G= \lambda [-{\nabla }^{2}\varphi +\varphi ({\varphi }^{2}-1)/{\varepsilon }^{2}$.

Surface tension parameter is directly proportional to the mixing energy density and inversely proportional to interface thickness $\sigma =2\sqrt{2}\lambda /3\varepsilon$^48^.

Apart from the standard boundary conditions including inlet and outlet, and wetted wall, the following boundary conditions exist on the walls:

7 $${\varvec{u}}=0$$

8 $$n.{\varepsilon }^{2}\nabla \varphi = {\varepsilon }^{2}\mathrm{cos}\theta \left|\nabla \varphi \right|$$

9 $$n.\left(\frac{\gamma \lambda }{{\varepsilon }^{2}}\right)\nabla \psi =0$$

where $\theta$ denotes contact angle. The Eq. (7) represents the no slip condition. The Eqs. (8) and (9) correspond to zero diffusive flux and change of total free energy on the surface respectively^48,52^.

### Variation of diffusive interface and fluid properties

The fluids properties of the $\text{CO}_{2}$ and Oil phases at specific temperature is represented in Table 3:

**Table 3:** Viscosity and density of pure $\text{CO}_{2}$ and oil phases in the system at constant temperature.

$$\rho_{{{\text{CO}}_{2} }} \left( {{\raise0.7ex\hbox{${{\text{kg}}}$} \!\mathord{\left/ {\vphantom {{{\text{kg}}} {{\text{m}}^{3} }}}\right.\kern-0pt} \!\lower0.7ex\hbox{${{\text{m}}^{3} }$}}} \right)$$	$$\mu_{{{\text{CO}}_{2} }} \left( {{\raise0.7ex\hbox{${\text{g}}$} \!\mathord{\left/ {\vphantom {{\text{g}} {{\text{m}}\;{\text{s}}}}}\right.\kern-0pt} \!\lower0.7ex\hbox{${{\text{m}}\;{\text{s}}}$}}} \right)$$	$$\rho_{oil} \left( {{\raise0.7ex\hbox{${{\text{kg}}}$} \!\mathord{\left/ {\vphantom {{{\text{kg}}} {{\text{m}}^{3} }}}\right.\kern-0pt} \!\lower0.7ex\hbox{${{\text{m}}^{3} }$}}} \right)$$	$$\mu_{oil} \left( {{\raise0.7ex\hbox{${\text{g}}$} \!\mathord{\left/ {\vphantom {{\text{g}} {{\text{m}}\;{\text{s}}}}}\right.\kern-0pt} \!\lower0.7ex\hbox{${{\text{m}}\;{\text{s}}}$}}} \right)$$	$$T({\text{K}})$$
319.9	0.0257	702.8	0.55726	344

The data of fluid density and viscosity are cited from http://webbook.nist.gov/chemistry/fluid/.

Gradually, by dissolving $\text{CO}_{2}$ moles into oil phase due to mass transfer effect, the properties of oil phase will be changed. The density and viscosity variation of the oil phase is calculated as a function of the concentration of dissolved $\text{CO}_{2}$ in the oil. Moreover, for the first time, dynamic variation is taken into account for interfacial tension and diffusivity coefficient as a function of pressure. All the corresponding correlations and explanations are presented in the Supporting information (section A).

### Mesh selection and numerical scheme

Triangular elements were used to resolve the domain. Finer mesh elements were selected for narrow channels and small pore throats while the coarser elements were used for pore bodies. To increase the accuracy of the model, at least 2 elements were used in narrowest throats.

**Figure 2:**
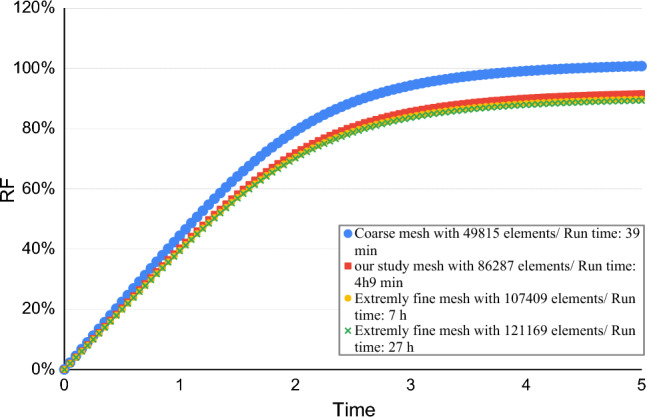
Mesh independence test for phase field model at near-miscible condition.

**Figure 3:**
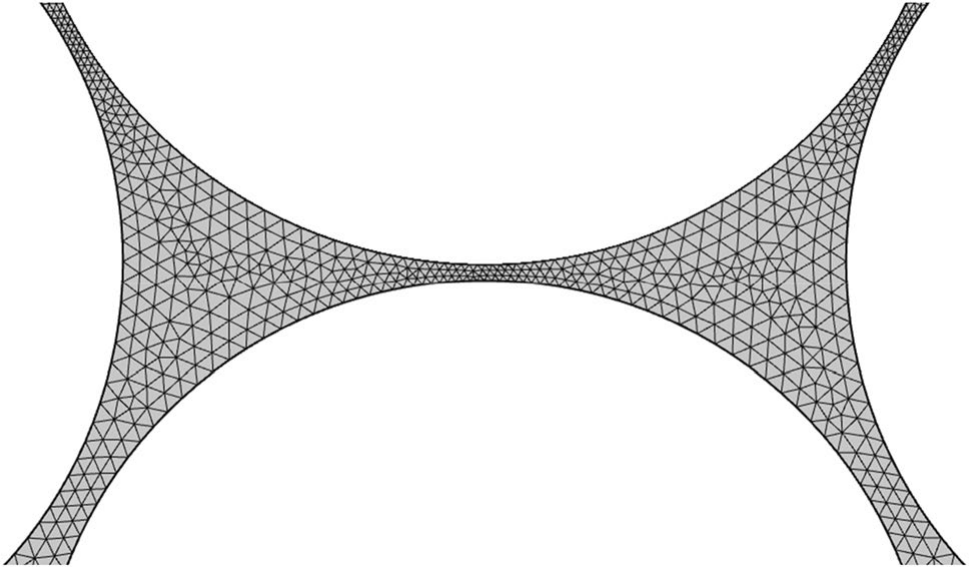
Triangular mesh elements in an enlarged section of the computational domain containing normal throats, narrow channels and pore bodies.

The related curves to the mesh independency based on case 1 as shown in the Fig. 2 is presented to predict the oil recovery coefficient (Fig. 3).

The recovery results change by increasing the number of meshes from 86,287 elements (in our study/fine mesh) to 107,419 (extra fine mesh) elements by only 2% and to 121,169 elements (extremely fine mesh) by only 2.5%, which changes with approximately 3 and 7 times the program execution time, respectively.

The finite element method (FEM) as numerical scheme is a popular method for numerically solving differential equations arising in engineering and mathematical modeling which is applied in this study.

The FEM is a general numerical method for solving partial differential equations in two or three space variables (i.e., some boundary value problems). To solve a problem, the FEM subdivides a large system into smaller, simpler parts that are called finite elements. This is achieved by a particular space discretization in the space dimensions, which is implemented by the construction of a mesh of the object: the numerical domain for the solution, which has a finite number of points. The finite element method formulation of a boundary value problem finally results in a system of algebraic equations. The method approximates the unknown function over the domain.

The implemented numerical model in this work was verified by analytical study of stratified two-phase Poiseuille flow^49,50^ and a perfect accuracy was reached.

## Results and discussion

This section presents the simulation results for the following three major cases:

1. Phase Field (PF) method in lower boundaries of effective near-miscible pressure region

The assumptions for this method are as follows:

- The interfacial tension between CO_2_ and oil is a function of pressure as presented in the supporting information (Section A)^53^.
- The changes in contact angle and wettability are small and could be ignored^54^. The contact angle is assumed to be $\theta =\frac{\pi }{6}$.
- The mode of injection is constant pressure at inlet.

2. Coupling/Combining Phase Field (PF) and Transport of Diluted Species Interface (TDS) processes in lower boundaries of effective near-miscible pressure region.

The assumptions for the PF+TDS method are as follows:

- CO_2_ diffusivity in oil is a function of pressure as presented in the supporting information (Section A).
- The interfacial tension between CO_2_ and oil is a function of pressure as presented in the supporting information (Section A)^53^.
- The changes in contact angle and wettability are small and could be ignored^54^. The contact angle is assumed to be θ = $\frac{\pi }{6}$.
- The mode of injection is constant pressure at inlet.

3. Ma et al.^43^ study (which used Phase Field (PF) method in lower boundaries of effective near-miscible pressure region).

Ma et al.’s assumptions are as follow:

- as CO_2_ diffusivity in oil is small (< 1 × 10*–*7 m2/s)^55^, CO_2_ diffusion into oil is very slow during immiscible and near-miscible flooding and can be reasonably ignored;
- The interfacial tension between CO_2_ and oil is almost constant^56^;
- The changes in contact angle and wettability are small and can be ignored^54^.
- The mode of injection is constant rate at inlet.

**Figure 4:**
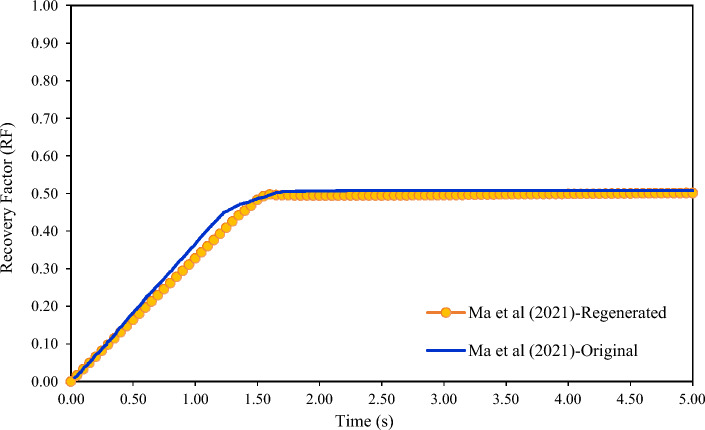
Recovery factor verification of this study and Ma et al^43^.

The simulation results of Cases 1 and 2 will be compared to Ma et al*.'s* (2021)^43^ results (Case 3) obtained using the PF method. Note that Ma et al*.'s* simulation results were regenerated with the relevant hypotheses and verified. The recovery factor curve, the most important curve obtained from the simulation, almost completely matches the graph from Ma et al*.'s* study which illustrated as Fig. 4.

### Changes in ${{\varvec{C}}{\varvec{O}}}_{2}$ saturation

**Figure 5:**
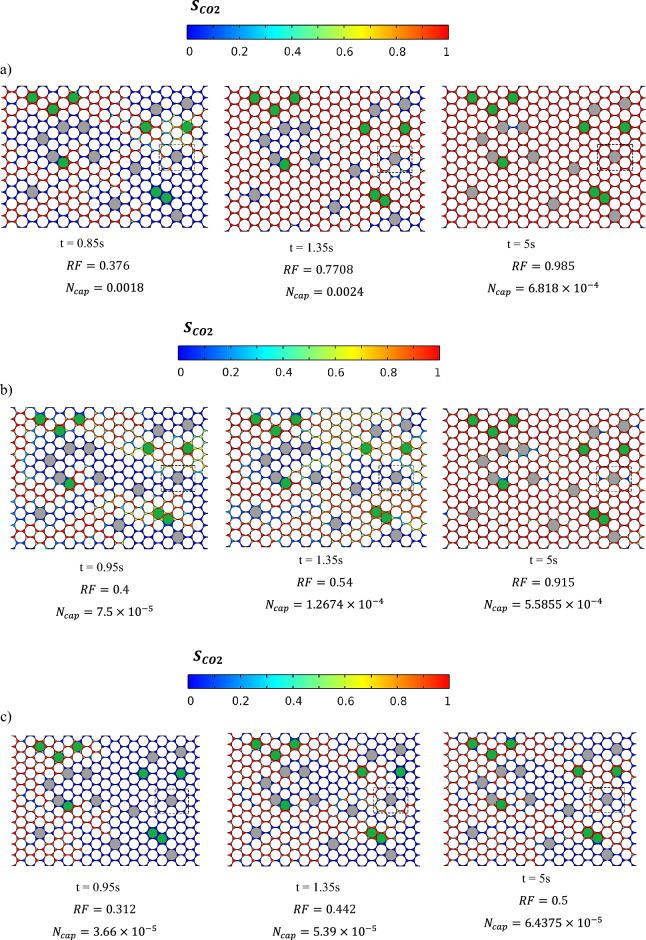
Temporal evolution of the calculated $\text{CO}_{2}$ saturation distribution under: (**a**) PF + TDS model. (**b**) PF model and (**c**) Ma et al. study.

Figure 5 shows gradual distribution changes in $\text{CO}_{2}$ saturation at breakthrough time, and end of simulation for the PF and PF + TDS cases. It should be noted that only for Ma et al*.'s* study a time equal to 0.95s before breakthrough is presented^43^.

The quantitative data are provided using the tools of color bars which are presented above for each subfigure and mainly the values of recovery factor (RF) in each timestep in Fig. 5.

This suggests that the PF + TDS case has better recovery factor than PF, both of which have significantly greater recovery factor than *Ma *et al*.'s*^43^ results, when comparing the end-of-simulation results.

Next, it could be clearly observed that at the same/similar time, the amount of $\text{CO}_{2}$ invasion and as a result, its concentration in the case of PF + TDS is significantly higher than the other two cases, so the breakthrough time for the case PF + TDS also occurred earlier than the other two cases.

For more information please refer to supporting information (Section C).

It should be noted that considering grains with three different sizes (small, normal, and large) in the pore structure of the model leads to heterogeneity, hence fingering emerges in the simulation results of the three mentioned cases.

The previous results and observations related to $\text{CO}_{2}$–oil saturation profiles can be analyzed and discussed with a cognitive mechanism in two topics.

**First topic** Pressure contour analysis across the pore-scale model for all time steps (from the initial time to the end of the simulation) while considering the model’s inlet and outlet pressure.

**Second topic** Residual oil saturation analysis for small to intermediate pore throats (created respectively by integrating one large grain with one normal grain or two normal grains).

### Pressure contour analysis

**Figure 6:**
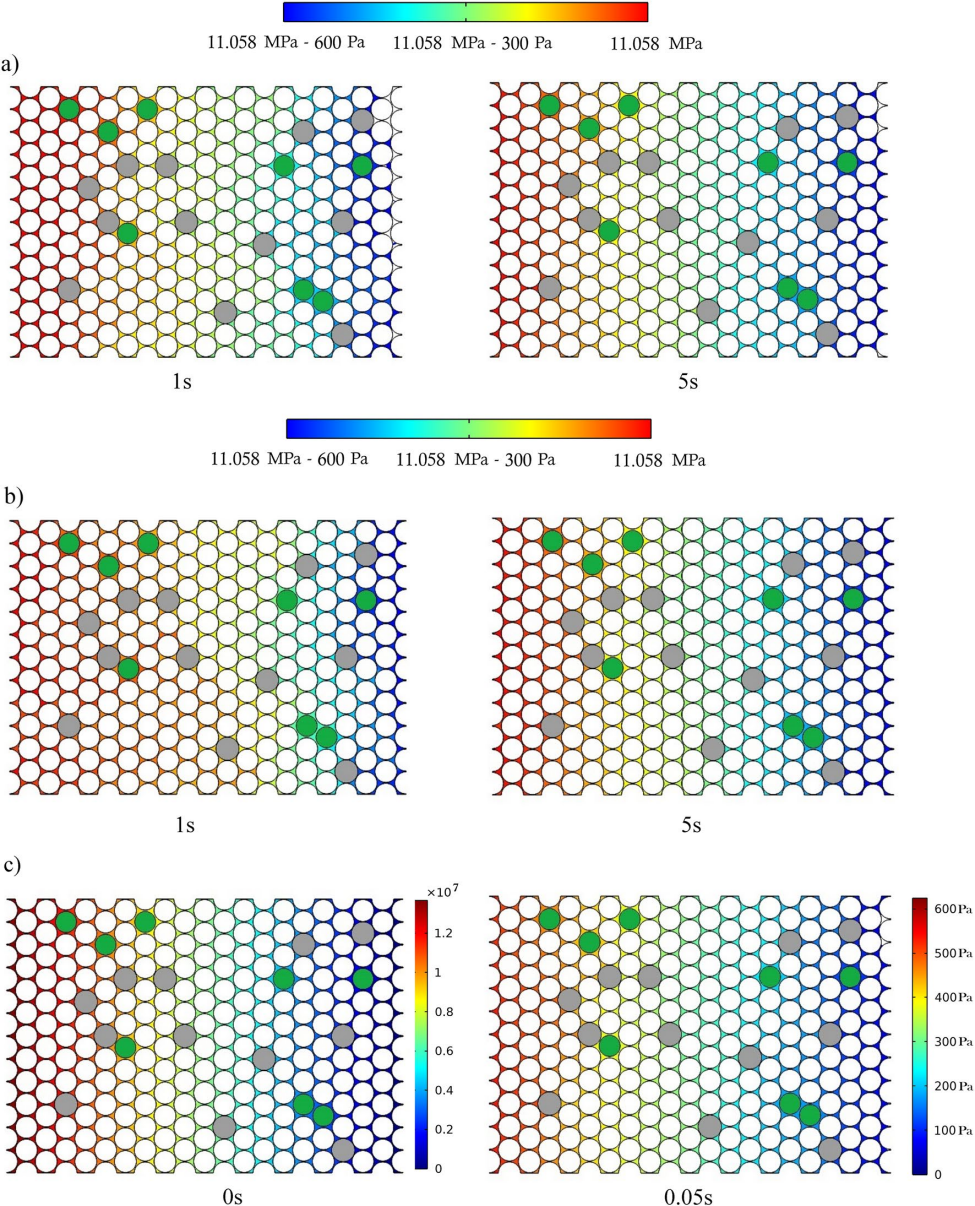
Pressure contour for all three cases under: (**a**) PF + TDS model, (**b**) PF model and (**c**) Ma et al.'s study.

Cases 1 and 2 were simulated by assuming a fixed inlet and outlet pressure boundary. According to Fig. 6a,b, the pressure difference is constant throughout the model and simulation (from the initial period until the end). The inlet and outlet pressures are at the lower boundaries of the effective near-miscible pressure region. Regarding Case 3, Ma et al*.* (2021) only considered the initial pressure in the lower boundary of the effective near-miscible pressure. According to Fig. 6c, applying an outlet pressure of zero places the pressure contour of the entire model in the immiscible region shortly after beginning of the simulation. Therefore, this simulation does not contain the minimum required pressure for the near-miscibility injection process.

### Residual oil saturation (in pore throat from small to normal sizes)

Applying the bundle of tube model to a porous medium based on the Hagen-Poiseuille equation indicates that the volumetric rate of the viscous flow varies in a specific pressure gradient according to the fourth power of pore radius^57,58^. According to first Fick’s law of diffusion, introducing mass transfer in a specific concentration gradient tie the volumetric rate of diffusion flow to the second power of the pore radius^59,60^.

Therefore, a relatively slight alteration in pore size/radius in pore-scale changes the volumetric flow rate of fluid through pores (due to viscous flow or diffusion term) by several orders of magnitude. Upon encountering pores of different radii (a heterogeneous medium) under identical conditions (same pressure gradient and concentration), the fluid prefers pores with larger radii ^61^. Thus, this section focuses on the part of the model with small-pore throats.

It is worth noting that although the surface tension is not zero in the near-miscible area, but due to the very low values of IFT in this area/range, the capillary forces will not be the dominant.

Log Cap—Log M (Capillary Number versus Viscosity Ratio) stability diagram showing three stability areas (bounded by dashed lines) and the locations of the $\text{CO}_{2}$ displacement simulated using PF and PF + TDS cases. The gray zones denote the stability areas indicated by Lenormand et al. ^62^.

**Figure 7:**
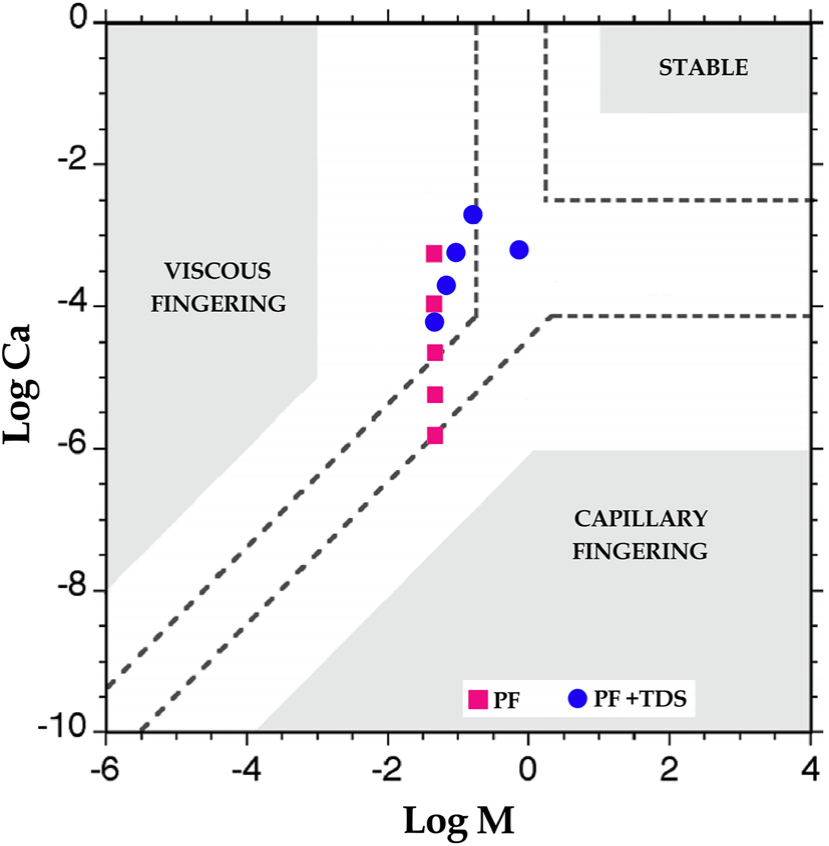
Capillary number versus viscosity ratio as stability diagram showing three stability areas (bounded by dashed lines) and the locations of the PF and PF + TDS results.

As shown in Fig. 7, due to mass transfer effect, the simulated data are moved from upper boundary of viscous fingering region to lower boundary of stable region which proves the capillary forces will not be the dominant.

So, the fluid flow behavior in pores (porous medium) will be a significantly stronger function of pore radius than capillary forces or IFT, due to following the governing equations of viscous flow and if considering the mass transfer term by following the governing equation of diffusion.

For each pore, there is a threshold capillary pressure for fluid entry based on the pore radius.

As discussed, due to gas injection in the near miscible region (and consequently low IFT values), the amount of this resistive pressure that prevents the gas entry into the pores occupied by the by-passed oil, is very small. Thus, more oil in pores comes into contact with the gas, and creates an effective driving force behind the main gas front ahead. Combined with mass transfer and the emergence of capillary cross flow, the main flow displaces the bypassed oil in pores (especially small to normal pores) toward the main flow.

When returning to the main flow, the transmissivity of oil is further enhanced by coupling it with the gas flow.

Studies by Williams & Dawe^63^, Jamiolahmady^64^, and Sohrabi & Danesh^28^ demonstrate the influence of simultaneous oil and gas flow in a specific pore in miscible displacement in near-miscible regions (very low IFT).

**Figure 8:**
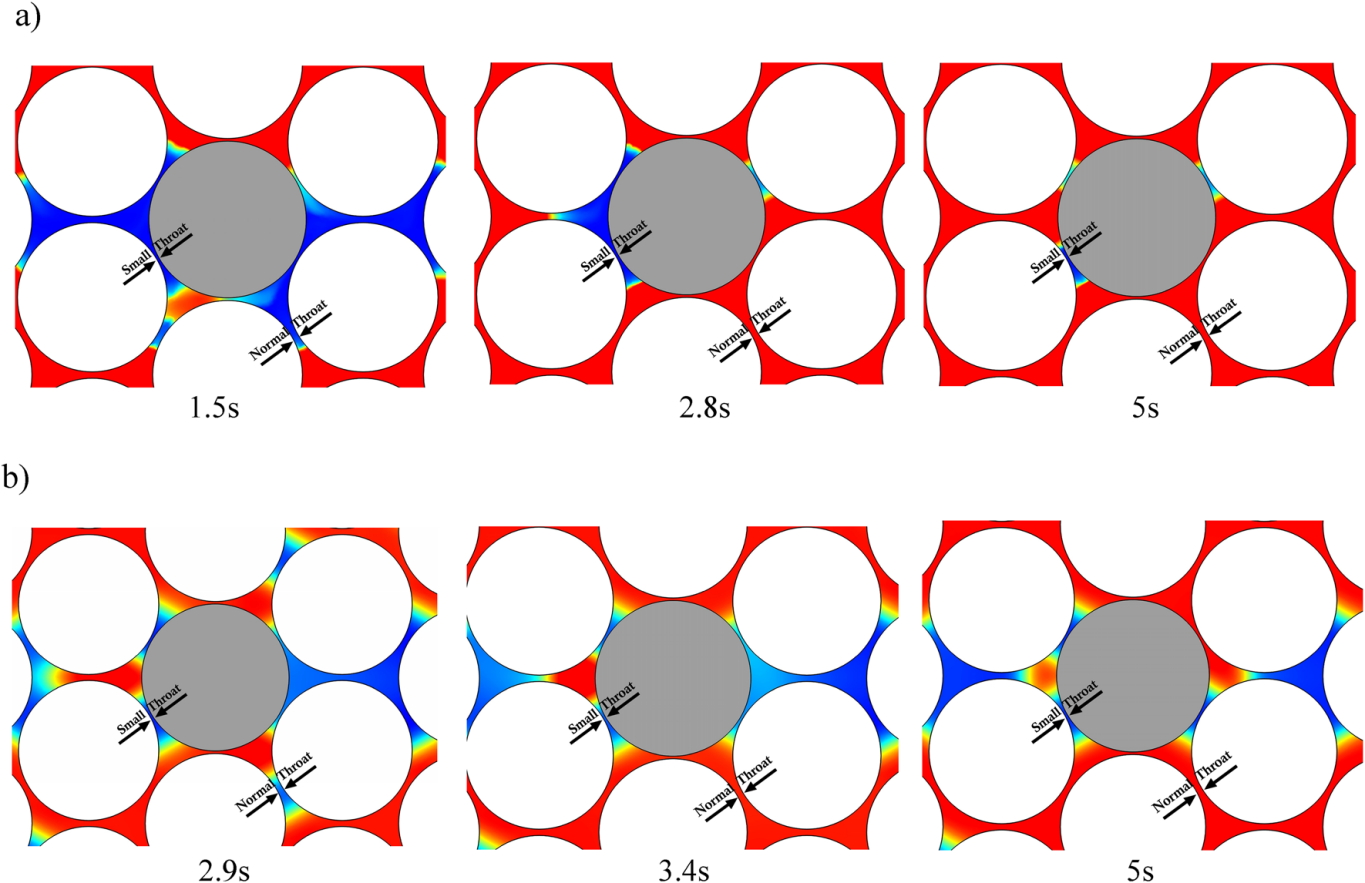
Specified part of pore-scale model during near-miscible $\text{CO}_{2}$ injection after the breakthrough of the $\text{CO}_{2}$ under: (**a**) PF + TDS model and (**b**) PF model.

For more accurate analysis, Fig. 8 represents residual (bypassed) oil in small to normal pore throats, after the breakthrough time, and the final simulation runtimes for Cases 1 and 2.

The influence of the diffusion term of volumetric flow rate (crossflow/mass transfer) in Case 2 (PF + TDS) compared to Case 1 (only PF), eventuates in almost zero saturation of residual (bypassed) oil in normal pore throats in Case 2, and very low saturation in small pore throats compared to Case 1, which indicates the near-complete recovery of oil related to Case 2.

This observation depicts the significance of considering the mass transfer term in gas injection modeling and simulation in the near-miscible front zone and its effect on sweeping residual oil, especially in small-pore throats.

The same mechanism that prevails over in the pores also applies to (semi) dead-end pores, which leads to an increase in the recovery of trapped oil in these pores.

**Figure 9:**
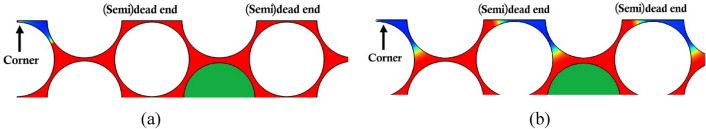
Enlarged upper part of the model in order to have better comparison about the (semi)dead-ends in both cases of (**a**) PF + TDS model and (**b**) PF model which indicates the performance of case a is significantly better than case 2.

According to Fig. 9a, there is near-zero residual oil in these pores at the end of the simulation run in Case 2 (PF + TDS); however, based on Fig. 9b significant residual oil in the same pores in Case 1 is detected (PF only). Figure 9 shows zero residual oil in the model's corners in both cases, albeit with slight differences.

The results are in good agreement with Sohrabi & Danesh (2008)^28^ and Seyyedi & Sohrabi (2020)^41^ in terms of evaluating oil recovery mechanisms under near-miscible conditions through injecting methane (first study) and $\text{CO}_{2}$ (second study) in a microfluidic chip saturated with a normal decane respectively.

Both studies confirm the strong cross flow phenomenon via mass transfer in pores occupied by bypassed oil. Hence, this phenomenon plays an unrivaled role in directing bypass oil in contact with gas to the main gas stream, which ultimately leads to an increase in almost fully oil recovery.

The by-passed oil recovery mechanism during the injection of near-miscible does not occur in immiscible and miscible injection.

Due to the intrinsic nature of the immiscible process which reflects high interfacial tension, a relatively stronger threshold capillary pressure is created at the oil–gas interface which prevents the simultaneous flow of oil and gas in the main flow. In this regard, studying the properties of phases will suffice for simulating immiscible flows which can be properly carried out using the PF method^42,43^.

Meanwhile, the miscible process has no oil–gas interface, and the system is fundamentally single-phase with no simultaneous oil and gas flow.

### Recovery factor

As mentioned earlier, given the model's heterogeneity, $\text{CO}_{2}$ first passes through the larger pores at the beginning of injection. Afterward, it rapidly moves toward the end of the model due to high mobility, bypassing a significant amount of oil. A short time after breakthrough, $\text{CO}_{2}$ first prefers to invade normal pores and then smaller pores due to mass transfer, a characteristic of the capillary crossflow. In this case, the diffusion phenomenon caused by mass transfer enhances the production of residual/trapped oil from these pores.

​Generally, by-passed oil phenomenon could emerge due to: undesirable mobility ratio, gravity override (if present), heterogeneities, dead-end pores, water (if present), and viscous fingering^28^.

**Figure 10:**
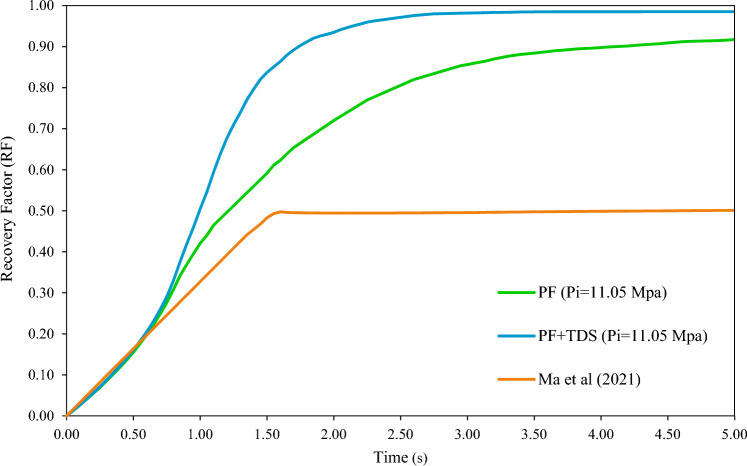
Oil recovery under PF + TDS and PF model in comparison with Ma et al.'s study.

According to Fig. 10, in addition to modeling two-phase flow displacement (oil and gas), recovery in Case 2 (PF + TDS) has also incorporated the effect of mass transfer (approximately 98.5%), which is 6% greater than Case 1 (PF only) that only simulated the movement of flowing phases. Hence, the recovery of Case 2 is much closer to the ideal final recovery of 100% obtained through the experimental study of miscible gas injection^65^ or a similar case in CFD-simulation of the porous medium^43^.In return to the simulation results of Case 3 (Ma's study) and given the fact that pressure throughout the model rapidly drops to very low levels (to immiscible regions), Fig. 9 shows that the recovery factor of Case 3 drops to a very low value 50% (throughout the model and simulation) in a near-49% drop compared to Cases 1 and 2 in this study.

The pore structure of this study is consistent with certain sandstone petroleum reservoirs. On the contrary to the relatively good inter-pore connectivity, this structure has a particular unavoidable heterogeneity^66^.

Miscible gas injection is often recommended for optimum recovery in the face of pore structure heterogeneity. However, achieving and maintaining miscibility conditions, mostly will be accompanied by associated operational difficulties and thus increased costs.

However, these results confirm that attaining an effective near-miscible pressure region throughout the model (porous medium) and providing an injection pressure near the miscible pressure can lead to near-maximum recovery. Therefore, to achieve maximum recovery during gas injection into heterogeneous reservoirs, gas injection at near miscibility pressure is recommended as an alternative solution that is more economical and feasible than gas injection at miscibility pressure^35^.

The results of numerous slim tube experiments using two-phase samples in the vapor–liquid equilibrium state show that reducing interfacial tension from a high value to near-zero (miscible condition) leads to near-zero irreducible oil saturation, and an increase of relative permeability (in a specified saturation). Ultimately, the relative permeability-saturation curves become almost straight diagonal lines. As a result, the case with low values of IFT or irreducible oil saturation is actually the condition of relative permeability-saturation curves under near-miscible conditions. Using various reservoir fluid samples, they found that one set of relative permeability-saturation curves obtained in a specific IFT is sufficient for interpreting the flow behavior of all fluid systems which have the same IFF value^67^.

The effect of IFT alteration on relative permeability-saturation curves in the gas phase is trivial. It is confirmed that phase transition doesn't essentially affect gas relative permeability, whereas the effect is significant on oil relative permeability. Therefore, Li et al. (2015)^68^ separately developed the exponential factor parameter based on Corey’s model as a piecewise function for immiscible, near-miscible, and miscible pressure regions.

Therefore, determining relative permeability-saturation curves and capillary pressure–saturation curves is crucial in controlling and checking gas–oil flow behavior during field-scale numerical simulation. Capillary pressure is known to follow IFT. As explained, relative permeability-saturation curves are also a function of interfacial tension, which is of great importance in near-miscible and miscible gas injection processes.

The pore-scale simulation results of this study suggest that when determining key parameters (related to field-scale) in near-miscible conditions, irreducible residual oil saturation and amount of capillary pressure must be lower than those used under immiscible injection. These values approach the miscible injection state in the limiting case where the capillary pressure is very small values (close to zero). This phenomenon is better apparent by considering the mass transfer term along with the movement of fluid phases.

### Pressure sensitivity analysis

It can be perceived that the pressure parameter (and consequently even the effective pressure region) to apply near-miscibility conditions throughout the porous media and its effect on alterations in the surface parameters, such as the surface tension parameter and mass transfer coefficient, is the foremost parameter in the sensitivity analysis in this study.

Hence, the model’s pressure in the lower boundaries of effective near-miscible pressure region increases from 0.87 MMP (11.05 MPa) to 0.9 MMP (11.5 MPa).

**Figure 11:**
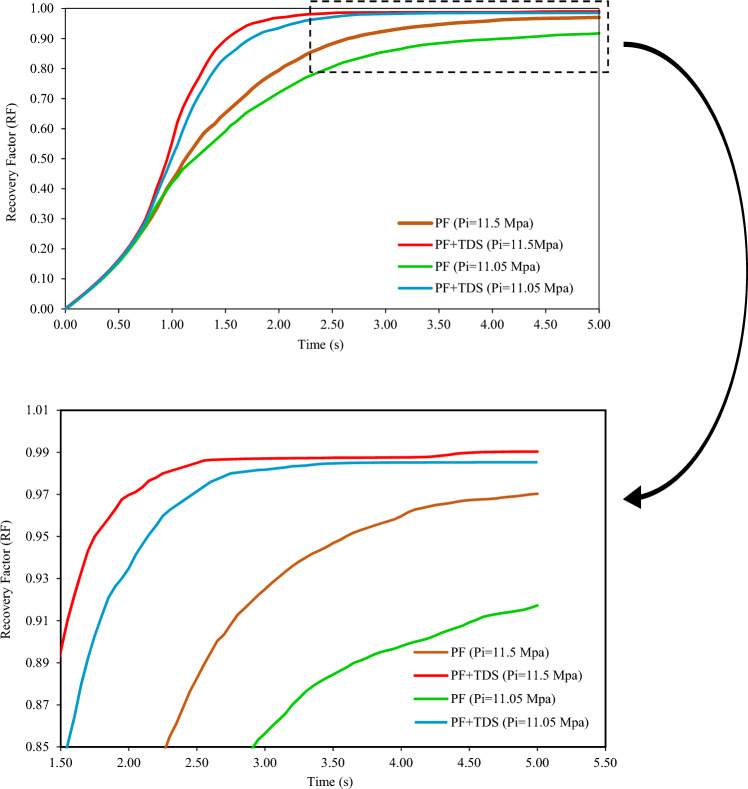
Oil recovery at the pressure of 11.05 Mpa and 11.5 Mpa, respectively, under PF + TDS and PF model.

Figure 11 shows the results obtained with the assumption of new and previous pressure in charts of oil recovery factor, suggesting that increasing pressure and approaching miscibility pressure generally increases oil recovery factor (both case 1 (PF only) and case 2 (PF + TDS)). Meanwhile, the greater recovery factor is significant in case 1 (PF only) and slight in case 2 (PF + TDS).

Note that even with greater pressure and the new pressure assumption, the oil recovery difference between case 1 (only PF) and case 2 (PF + TDS) and the effect of mass transfer are evident.

In a way, this result confirms that including the lower limit of effective near-miscible pressure region (more specifically, the lowest possible pressure in this region) and also the mass transfer term in pore-scale modeling (based on governing mechanism explained in the near-miscible region), same oil recovery factor could be achieved with a slight difference and lower cost.

## Conclusion

In this study, a numerical simulation approach has been carried out in order to perceive the flow behavior and the displacement mechanism of $\text{CO}_{2}$–Oil at the pore scale, under the near-miscible condition in a heterogeneous porous medium.

The following conclusion can be found out:

- Using this approach, it is discerned that for both PF & PF + TDS cases, $\text{CO}_{2}$ preferably displaces oil through big throats, while for PF + TDS as a consequence, invades (normal to) small pore throats, which considerably increases oil recovery efficiency.
- Strengthening $\text{CO}_{2}$ diffusion for flooding under an effective near-miscibility region is preferred for oil reservoirs with a wide range of pore structures. This process makes oil displacement by near-miscible gas flooding a recommended method.
- Pressure changes in the near-miscible region and results of sensitivity analysis illustrate that the greater pressure in PF + TDS modeling has not significantly influenced oil recovery, which suggests that considering the effect of mass transfer in modeling has increased oil recovery toward the feasible maximum, thereby addressing the increase in operating costs.

We propose the work outcomes to apply in other fields such as displacement water/oil or cushion gas during geological hydrogen storage.

### Supplementary Information

Supplementary Information.

## Data Availability

All data generated or analyzed during this study are included in this published article. It will be available upon request. The corresponding author (MRK) should be contacted for this purpose.

## List of symbols

$$\text{CO}_{2}$$: Carbon dioxide
IFT: Interfacial tension
EOR: Enhanced oil recovery
FEM: Finite element method
T: Temperature
Cap: Capillary number
P: Pressure
C: Concentration of $\text{CO}_{2}$ phase
$${\rho }_{oil}$$: Oil density
$${\mu }_{oil}$$: Oil viscosity
$${P}_{inj}$$: Injection pressure
$${P}_{out}$$: Outlet pressure
$$\sigma$$: Interfacial tension
$${S}_{\text{CO}_{2}}$$: $$\text{CO}_{2}$$ saturation
$$G$$: Chemical potential
$\varepsilon$: Thickness of the interface
$$\psi$$: Auxiliary parameter
TDS: Transport of diluted species interface
PF: Phase field
IOR: Improved oil recovery
MMP: Minimum miscibility pressure
t: Time
M: Viscosity ratio
$u$: Fluid velocity field
$$D$$: Diffusion coefficient
$${\rho }_{\text{CO}_{2}}$$: $$\text{CO}_{2}$$ density
$${\mu }_{\text{CO}_{2}}$$: $$\text{CO}_{2}$$ viscosity
$${P}_{init}$$: Initial pressure
$$\theta$$: Contact angle
${S}_{\text{CO}_{2}, ave}$: Average saturation of $\text{CO}_{2}$
$${N}_{cap}$$: Capillary number
$\varphi$: Relative concentration of each phase
$\lambda$: Mixing energy density
*γ*: Mobility parameter
