# Supplementary material for: Numerical analysis of pore-scale CO2-EOR at near-miscible flow condition to perceive the displacement mechanism
Source: Sci Rep. 2023 Aug 3;13:12632. doi: 10.1038/s41598-023-39706-1 (PMC10400605; doi:10.1038/s41598-023-39706-1)
Supplement: Supplementary file 1 — Supplementary Information. [file 41598_2023_39706_MOESM1_ESM.docx]

**Numerical Analysis of Pore-Scale CO2-EOR at Near-Miscible Flow Condition to Perceive the Displacement Mechanism**

Parisa Behnoud ^1^, Mohammad Reza Khorsand Movaghar ^1, *^, Ehsan Sabooniha ^1, 2^

^1^ Department of Petroleum Engineering, Amirkabir University of Technology (Tehran Polytechnique), 424 Hafez Avenue, Tehran, Iran, 1591634311, P.O. Box 15875-4413

^2^ Technical University of Denmark (DTU), PhD Candidate at Danish Offshore Technology Center

* Corresponding author: E-mail: [m.khorsand@aut.ac.ir](mailto:m.khorsand@aut.ac.ir), Tel.: +98 21 64545133

1^st^ author email: [parisabehnoud@aut.ac.ir](mailto:parisabehnoud@aut.ac.ir), 3^rd^ author email: e.sabooniha@aut.ac.ir

**Supplementary File**

1. Variation of diffusive interface and fluid properties

Gradually, by dissolving ${CO}_{2}$ moles into oil phase due to mass transfer effect, the properties of oil phase will be changed. The density variation of oil phase is calculated with the following linear equation **[1,2]**:

| $\rho= \rho\left( c \right)=\frac{C - C_{0}}{C_{inj} - C_{0}}\rho_{{CO}_{2}}+(1-\frac{C - C_{0}}{C_{inj}- C_{0}})\rho_{oil}$ | ( 1) |
| --- | --- |

The viscosity variation of oil phase will be calculated by a quarter-law mixing rule **[3-5]**:

| $\mu= \mu\left( c \right)= {[\frac{C - C_{0}}{C_{inj} - C_{0}}\mu_{{CO}_{2}}^{-0.25}+\left( 1-\frac{C - C_{0}}{C_{inj}- C_{0}} \right)\mu_{oil}^{-0.25}]}^{-4}$ | ( 2) |
| --- | --- |

In equation (2), $c$ denotes the concentration of dissolved ${CO}_{2}$ in the oil phase $c_{inj}$is defined by the value of inlet concentration of dissolved ${CO}_{2}$ in oil which is specified as$1.5 \frac{mol}{m^{3}}$ , $c_{0}$is the initial concentration of ${CO}_{2}$ in the domain which is equal to zero.

There is an imbalance of molecular forces at interface between two phases. This is caused by physical attraction between molecules. This imbalance of force is known as interfacial tension (IFT). The rational fitting model was used to calculate the dynamic variation of IFT with pressure using the data points (Fig. 1-a) presented in the Shaver. et al work**[6]**:

| $\sigma= \sigma\left( c \right)=\frac{a+bp}{a+cp+dp^{2}}$ | ( 3) |
| --- | --- |

Where p is the pressure in (*Mpa*) and a, b, c, d constants are 19.68, -1.55, 0.005, and 0.002 respectively. The data points presented in Bagalkot. et al work **[7]** (Fig. 1-b) was used to calculate diffusion coefficient variation for pressure over 9 Mpa and the following equation was derived using sigmoidal fitting model:

| $D=D\left( c \right)= \frac{a}{{(1+e^{b-cp})}^{\frac{1}{d}}}$ | ( 4) |
| --- | --- |

Where p is the pressure in (bar) and a, b, c, d are constant with 0.41, 53.78, 0.38, and 58.49 values.

| 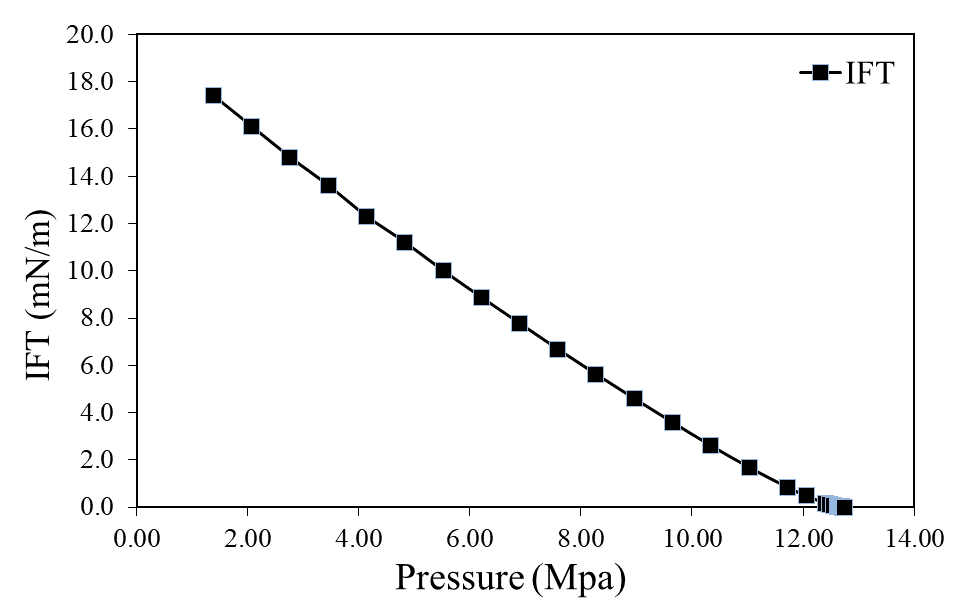 (a) | 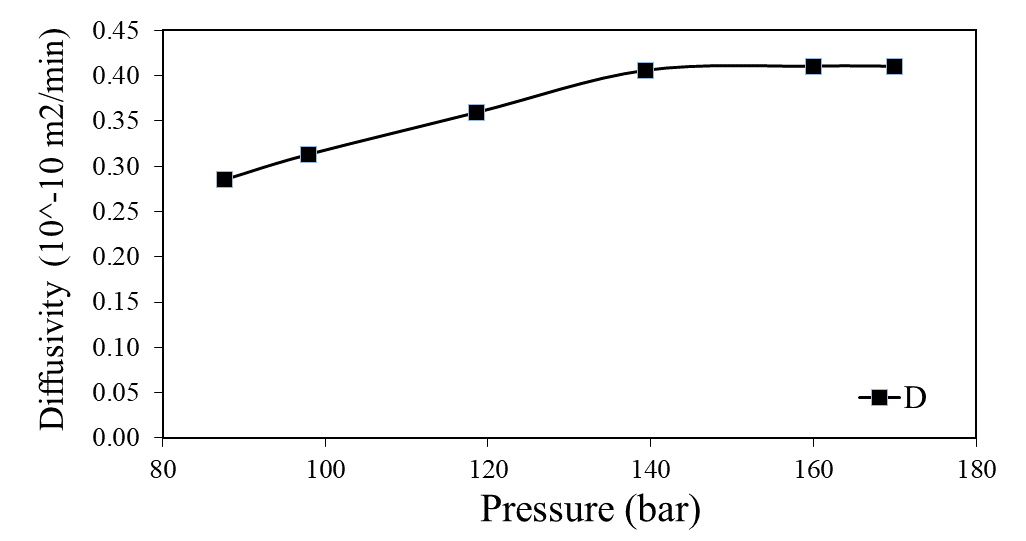 (b) |
| --- | --- |

Fig 1. (a) The pressure based IFT variation **[8]**. (b) Diffusion coefficient variation for different pressures **[9]**.

1. Homogenous and Heterogenous model geometry

It should be noted that snapshots from the heterogenous media (small pore throat/ corresponding to normal to large grain) at the end time (5 seconds) of PF+TDS and PF models have been previously presented in figure 8.a and 8.b.

However, to distinguish the effect of carbon dioxide displacement in homogeneous and heterogeneous porous media, snapshots from the homogeneous (only normal grains in this study) and heterogenous medium at the end time (5 seconds) of PF+TDS and PF models have been provided and presented as follows.

| 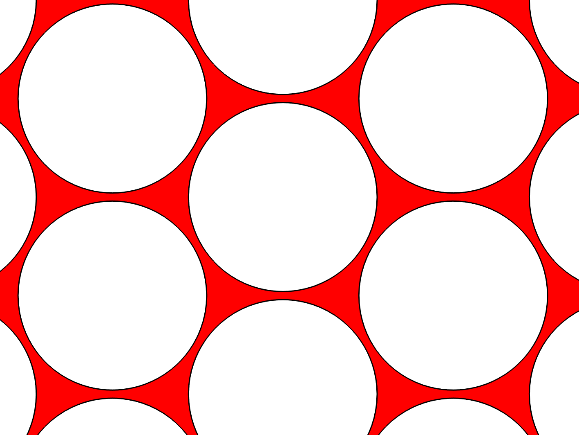  (a) | 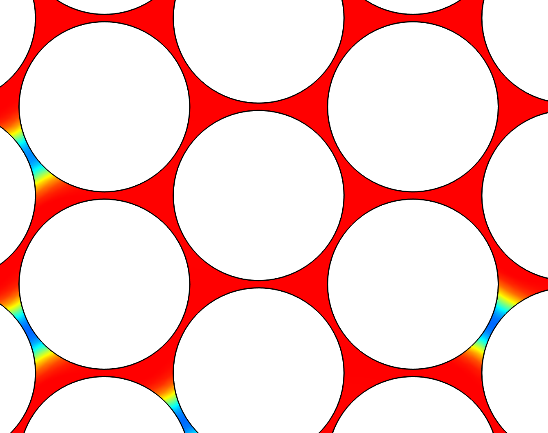  (b) |
| --- | --- |

Fig 2.Specified part of pore-scale model during near-miscible ${CO}_{2}$ injection at t=5s under a) PF+TDS model and b) PF for homogenous geometry model.

| 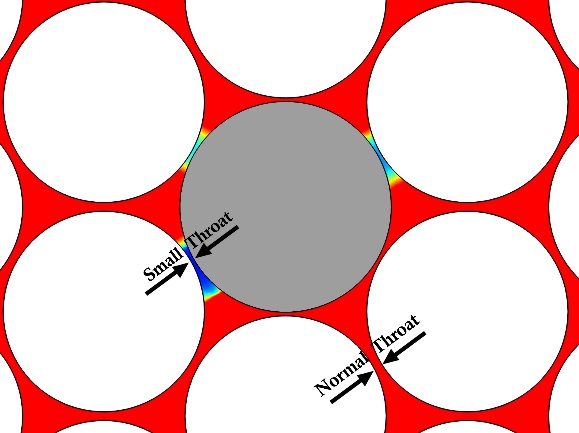 (a) | 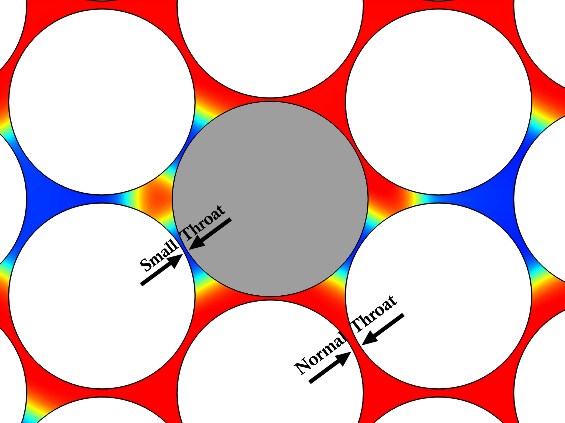 (b) |
| --- | --- |

Fig 3.Specified part of pore-scale model during near-miscible ${CO}_{2}$ injection at t=5s under a) PF+TDS model and b) PF for heterogenous geometry model.

Comparing the PF model’s end results for the homogeneous and heterogeneous media clearly shows that the former has lower residual saturated oil than the latter (according to subfigure 8-b). Despite modeling in near-miscible pressure conditions, there is significant residual oil entrapment in small to normal pores due to ignoring the mass transfer effect of the gas injected into the oil decane and the heterogeneous structure.

Now, comparing the end time for the homogeneous and heterogeneous porous media using the PF+TDS model clearly shows zero residual oil saturation for the former and non-zero residual oil saturation for the latter (subfigure 8-a). As explained in the study, residual oil has been trapped in the small pore throats resulting from the heterogeneous medium’s structure.

In other words, using the PF+TDS model to apply the mass transfer effect results in zero residual oil saturation in the homogenous porous medium. However, the heterogeneous structure and the creation of narrow regions will leave some residual oil saturation in small pores, albeit much lower than the PF model.

1. Changes in $\mathbf{CO}_{\mathbf{2}}$ Concentration

Since to simulate case 2, the TDS module has been coupled to the PF module, hence capturing the capability of the TDS it could be possible to illustrate the concentration profile of dissolved$\mathrm{CO}_{2}$ in oil throughout the porous medium. Fig 4. shows gradual distribution changes in$\mathrm{CO}_{2}$ concentration at breakthrough time, 1.35 s, and end of simulation for the PF+TDS case.

| 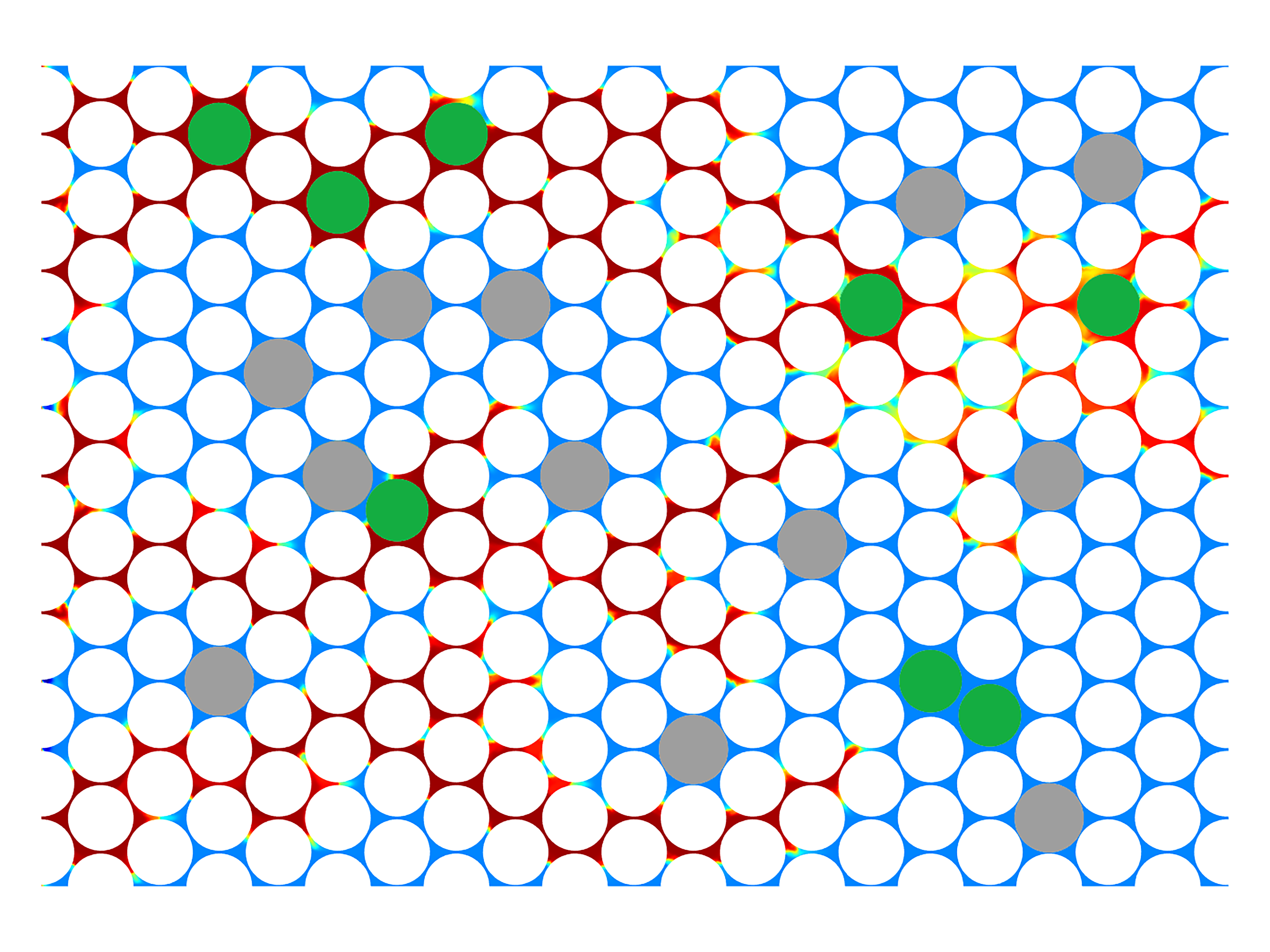 t=0.85s | 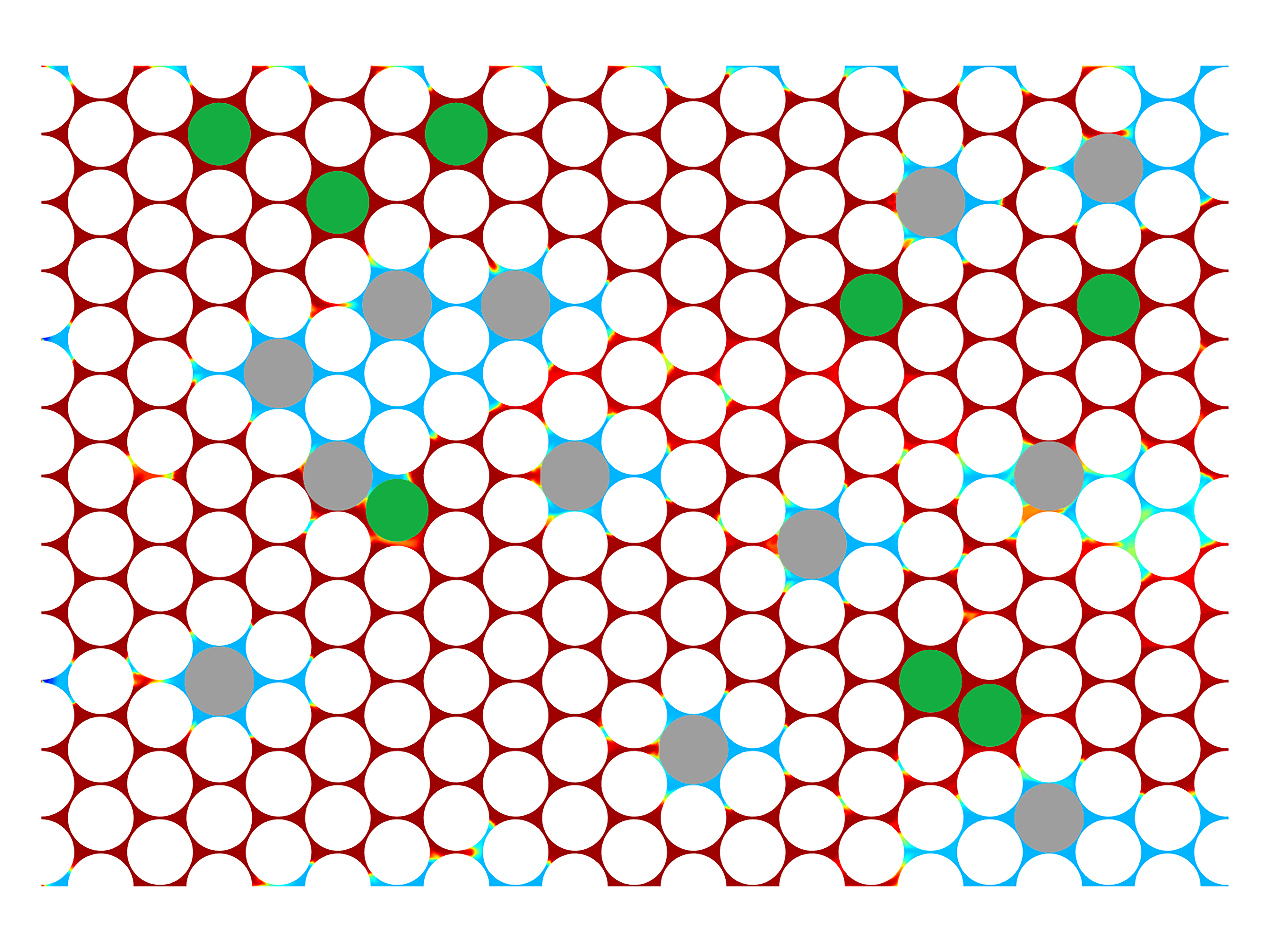 t=1.35s | 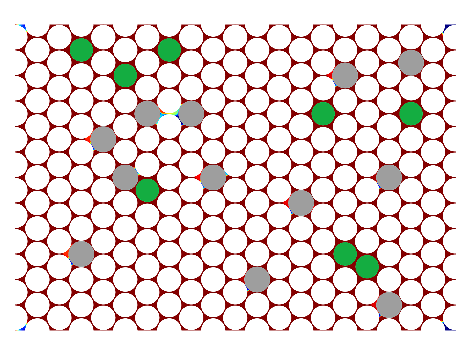  t=5 s |
| --- | --- | --- |

Fig 4.Temporal evaluation of the calculated distribution of dissolved ${CO}_{2}$ concentration in oil under PF+TDS model

**Reference:**

1 Dumore, J. Stability considerations in downward miscible displacements. *Society of Petroleum Engineers Journal* **4**, 356-362 (1964).

2 Tan, C. & Homsy, G. Stability of miscible displacements in porous media: Radial source flow. *The Physics of fluids* **30**, 1239-1245 (1987).

3 Koval, E. A method for predicting the performance of unstable miscible displacement in heterogeneous media. *Society of Petroleum Engineers Journal* **3**, 145-154 (1963).

4 Stalkup, F. Miscible Displacement, Monograph Volume 8, Henry L. *Doherty Series, SPE, Richardson, Texas* (1983).

5 Afshari, S., Hejazi, S. H. & Kantzas, A. Role of medium heterogeneity and viscosity contrast in miscible flow regimes and mixing zone growth: A computational pore-scale approach. *Physical Review Fluids* **3**, 054501 (2018).

6 Shaver, R., Robinson Jr, R. & Gasem, K. An automated apparatus for equilibrium phase compositions, densities, and interfacial tensions: data for carbon dioxide+ decane. *Fluid phase equilibria* **179**, 43-66 (2001).

7 Carman, P. C. Fluid flow through granular beds. *Chemical Engineering Research and Design* **75**, S32-S48 (1997).

8 AlMubarak, T. *et al.* in *SPE Saudi Arabia Section Annual Technical Symposium and Exhibition.* (OnePetro).

9 Bagalkot, N. & Hamouda, A. A. Interfacial tension and CO 2 diffusion coefficients for a CO 2+ water and n-decane system at pressures of 10 to 160 bar. *RSC advances* **8**, 38351-38362 (2018).

**Figure legends**

Fig 1. (a) The pressure based IFT variation **[8]**. (b) Diffusion coefficient variation for different pressures **[9]**.

Fig 2. Specified part of pore-scale model during near-miscible $\mathrm{CO}_{2}$ injection at t=5s under a) PF+TDS model and b) PF for homogenous geometry model.

Fig 3. Specified part of pore-scale model during near-miscible$\mathrm{CO}_{2}$ injection at t=5s under a) PF+TDS model and b) PF for heterogenous geometry model.

Fig 4. Temporal evaluation of the calculated distribution of dissolved $\mathrm{CO}_{2}$ concentration in oil under PF+TDS model
